# Mutation profiling of anaplastic ependymoma grade III by Ion Proton next generation DNA sequencing

**DOI:** 10.12688/f1000research.18721.2

**Published:** 2020-06-22

**Authors:** Ejaz Butt, Sabra Alyami, Tahani Nageeti, Muhammad Saeed, Khalid AlQuthami, Abdellatif Bouazzaoui, Mohammad Athar, Zainularifeen Abduljaleel, Faisal Al-Allaf, Mohiuddin Taher

**Affiliations:** 1 Histopathology Division, Al-Noor Specialty Hospital, Makkah, Makkah, Saudi Arabia; 2 Histopathology Department, Amna Inayat Medical College, Sheikhupura, Punjab, Pakistan; 3 Department of Medical Genetics, Umm-Al-Qura University, Makkah, Makkah, Saudi Arabia; 4 Department of Radiation Oncology, King Abdullah Medical City, Makkah, Makkah, Saudi Arabia; 5 Faculty of Medicine, Umm-Al-Qura University and Al-Noor Specialty Hospital, Makkah, Makkah, Saudi Arabia; 6 Department of Laboratory Medicine and Blood Bank, Al-Noor Specialty Hospital, Makkah, Makkah, Saudi Arabia; 7 Department of Medical Genetics and Science and Technology Unit, Umm-Al-Qura University, Makkah, Makkah, Saudi Arabia

**Keywords:** Ependymoma, Palisading necrosis, Perivascular psuedorossettes, Ion Proton, Next Generation DNA sequencing, Glioma, pediatric brain tumors, Anaplastic Ependymoma

## Abstract

**Background: **Ependymomas are glial tumors derived from differentiated ependymal cells. In contrast to other types of brain tumors, histological grading is not a good prognostic marker for these tumors. In order to determine genomic changes in an anaplastic ependymoma, we analyzed its mutation patterns by next generation sequencing (NGS).

**Methods:  **Tumor DNA was sequenced using an Ion PI v3 chip on Ion Proton instrument and the data were analyzed by Ion Reporter 5.6.

**Results: **NGS analysis identified 19 variants, of which four were previously reported missense variants; c.395G>A in *IDH1*, c.1173A>G in *PIK3CA*, c.1416A>T in *KDR* and c.215C>G in *TP53*. The frequencies of the three missense mutations ( *PIK3CA* c.1173A>G, *KDR* c.1416A>T, *TP53*, c.215C>G) were high, suggesting that these are germline variants, whereas the *IDH1* variant frequency was low (4.81%). However, based on its FATHMM score of 0.94, only the *IDH1* variant is pathogenic; other variants *TP53*, *PIK3CA* and *KDR* had FATHMM scores of 0.22, 0.56 and 0.07, respectively. Eight synonymous mutations were found in *FGFR3*, *PDGFRA*, *EGFR*, *RET*, *HRAS*, *FLT3*, *APC* and *SMAD4* genes. The mutation in *FLT3* p.(Val592Val) was the only novel variant found. Additionally, two known intronic variants in *KDR *were found and intronic variants were also found in *ERBB4* and *PIK3CA*. A known splice site mutation at an acceptor site in *FLT3*, a 3’-UTR variant in the *CSF1R* gene and a 5’_UTR variant in the *SMARCB1* gene were also identified. The p-values were below 0.00001 for all variants and the average coverage for all variants was around 2000x.

**Conclusions: **In this grade III ependymoma, one novel synonymous mutation and one deleterious missense mutation is reported. Many of the variants reported here have not been detected in ependymal tumors by NGS analysis previously and we therefore report these variants in brain tissue for the first time.

## Introduction

Ependymal cells are macroglial cells which line the ventricles, the central canal of the spinal cord and form the blood-cerebrospinal fluid barrier, being involved in producing the cerebrospinal fluid ^1, 2^. These tumors account for only 4–8% of gliomas and, after astrocytomas and oligodendrogliomas, ependymomas are the least common ^3^. Nearly one-third of brain tumors in patients younger than three years old are ependymomas and constitute around 5%–9% of all neuroepithelial malignancies ^1, 4^. These tumors are also found in the choroid plexus and may occur at any age, from one month to 81 years and without any gender preference ^5^. In pediatric cases, the location of the tumor is intracranial, while adult ependymal tumors can have either an intracranial or a spinal localization ^6, 7^. The prognosis is better in older children as compared to young infants but nonetheless, in children with intracranial ependymomas, event-free survival after five years is less than 50% ^8^. In adults, about 50% to 60% intracranial ependymomas are supratentorial; however, pediatric supratentorial ependymomas account for 25% to 35% of all ependymomas ^5, 9^. Adults present better prognosis with a 5-year survival of around 90%, while in the pediatric population it is around 60%. The five-year survival rate for supratentorial, infratentorial, and spinal cord ependymomas is 62%, 85%, and 97%, respectively, and for grade I, II, and III spinal cord ependymomas the five-year overall survival rate is 92%, 97% and 58%, respectively ^10– 12^.

Ependymoma tumors are well circumscribed, soft, tan-red masses and may be associated with hemorrhage. Their microscopic appearance shows hypercellularity and distinct infiltrative margins with surrounding parenchyma, consisting of monomorphic cells with nuclear atypia and brisk mitotic activity. They may also have intramural or glomeruliod vascular proliferation, pseudopalisading necrosis, perivascular pseudo rosettes (5–10% cases), calcifications and hyalinized vessels ^1^. Other diagnostic hallmarks include areas of fibrillary and regressive changes such as myxoid degeneration, palisading necrotic areas and the formation of true rosettes, composed of columnar cells arranged around a central lumen ^1, 6^. Immunologically, they are positive for epithelial membrane antigen (EMA), glial fibrillary acidic protein (GFAP) and S-100. According to the 2016 updated World Health Organization (WHO) classification of brain tumors, ependymomas are divided into 4 types on the basis of histologic appearance: (1) grade I subependymomas, (2) grade I myxopapillary ependymomas, (3) grade II ependymomas, (4) grade II or III *RELA* fusion-positive ependymomas and grade III anaplastic ependymomas ^13, 14^.

Previous studies have shown the use of comparative genomic hybridization (CGH) arrays to distinguish intracranial ependymomas from spinal ependymomas ^15^. In contrast to other types of brain tumors, histological grading is not a good prognostic marker for outcome for ependymomas ^16, 17^. Several gene expression studies have been helpful in differentiating between intracranial and extra cranial ependymomas, but have not had clinical significance in directing therapy and their role in tumor origin and prognosis is not clear ^18, 19^. Studies using cDNA micro-arrays have shown that gene expression patterns in ependymomas correlate with tumor location, grade and patient age ^20^. Cytogenetic studies have shown that chromosomal abnormalities are relatively common in ependymomas ^21^. Loss of 22q has been the commonest abnormality found in ependymoma and, in some other tumors, gain of 1q or loss of 6q was observed ^21, 22^.

To date, there is a lack of information regarding the mutational signatures which distinguish the various subgroups of ependymomas. Another ependymoma cohort study found very few mutations and gene amplifications but a high expression of multi-drug resistance, DNA repair and synthesis enzymes ^23^. Intracranial ependymomas differ from spinal ependymomas in the expression of these proteins, and protein expression is also dependent on the ependymoma grade ^23^. For both intracranial and spinal ependymomas, very few mutations were reported by using whole exome sequencing ^24^. In another study, profiling of NGS mutations was carried out for one case of grade II ependymoma using a GlioSeq panel, which contains a total of 30 genes ^25^. In order to determine the mutational patterns of grade III anaplastic ependymoma, we have sequenced DNA from this ependymoma tumor using the Ion Proton system for next generation DNA sequencing with the Ion Torrent’s AmpliSeq cancer HotSpot panel. This panel contains 50 genes, only 15 of which also appear in the GlioSeq panel used in previous research. These data provided an evaluation of mutational signatures of this anaplastic ependymoma which differs from the previous two studies, but confirms their conclusions about finding very few mutations in cancer driver genes, helping to direct diagnosis and therapy for ependymomal tumors.

## Methods

### Ethical statement

This study was performed in accordance with the principles of the Declaration of Helsinki. This study was approved by the Institutional Review Board (IRB) bioethics committee of King Abdullah Medical City (KAMC), Makkah, Kingdom of Saudi Arabia (IRB number 14-140). A written informed consent was obtained from the parent of this patient before starting the study.

### Clinical specimen

The single patient’s tumor tissue (FFPE sections in PCR tubes) used in this NGS analysis was obtained from the histopathology laboratory of Al-Noor Specialty Hospital Makkah, after tumor excision and left frontal craniotomy in the neurosurgery department. The tumor content of the FFPE tissues was around 70–80%. The tumor was classified based upon similarity to the constituent cells of the central nervous system, such as astrocytes, oligodendrocytes and ependymal, glial cells, mitosis and cell cycle-specific antigens, used as markers to evaluate proliferation activity and biological behavior (the WHO grading system) ^13^. The final diagnosis was made following radiological, histopathological and immunological examinations.

### Radiology and histopathological analysis

A CT scan of the brain was performed by a multi-slice CT (MSCT), using a 64-detector-row scanner. The use of computed tomography (CT) allowed visualization of detailed images of the soft tissues in the body in 3D as well as in multiplanar reconstructions. Images were acquired with 5mm slice thickness throughout on a GE Medical Systems, light speed VCT, 64-slice multidetector CT (MDCT). High quality images were processed at low dose performance on Volara™ digital DAS (Data Acquisition System).

The excised tumor was fixed in 4% buffered formaldehyde, routinely processed and paraffin embedded. Four-micrometer-thick sections were prepared on clear ground glass microscope slides with ground edges and routinely stained using Dako Reagent Management System (DakoRMS) with hematoxylin and eosin (H and E) on a Dako Coverstainer (Agilent). For immunohistochemistry, sections were collected on Citoglas adhesion microscope slides ( Citotest). Mouse monoclonal beta-catenin (14) (Sigma-Aldrich, cat. no. 224M-1), mouse monoclonal EMA (E29) (Sigma-Aldrich, cat. no. 247M-9), rabbit monoclonal EGFR (SP84) (Cell Marque, cat. no. 414R-16-ASR), mouse monoclonal Vimentin (vim 3B4) (Ventana-Roche, cat. no. 760-2512), GFAP EP672Y rabbit monoclonal (Ventana-Roche, cat. no. 760-4345) and E-cadherin (36) mouse monoclonal (Ventana-Roche, cat. no. 790-4497) and mouse monoclonal anti-Ki-67 (Leica Biosystems, cat. no. KI67-MM1-L-CE) antibodies were used for immunohistochemistry. Briefly, the tissue sections were deparaffinized with EZ Prep (Ventana, cat. no. 950-102) at 60°C for 1 hr. Immunohistochemistry was performed with the Ventana BenchMark XT automated stainer (Ventana, Tucson, AZ). After inactivation of the endogenous peroxidase using a UV-inhibitor for 4 min at 37°C, the primary antibody was added for 16 min at 37°C, followed by the application of HRP Universal Multimer for 8 min, and detected using the ultraView Universal DAB Detection Kit (cat. no. 760-500) for 38 min. Slides were counterstained with hematoxylin for 8 min and bluing reagent for 4 min before mounting with cover slips. Following staining, images were acquired using NIKON Digital Microscope Camera - DS-Ri1, with image software NIS Elements v.4.0. Appropriate positive controls for all of the studied antibodies were used.

### DNA isolation and NGS analysis

DNA isolation was carried out using the QIAamp DNA FFPE Kit (50), Cat. No. 56404. 5-10 Formalin-Fixed Paraffin-Embedded sections of 5 microns were deparaffinized using xylene, treated with ethanol to remove the xylene, and the pellet was dried at 65°C for 5 mins. The pellets were resuspended in ATL buffer then treated with proteinase K. The remaining steps were carried out according to the user manuals. DNA concentration was measured using Nanodrop2000C and 10 ng of DNA was used for NGS analysis. DNA was sequenced using the Ion PI v3 Chip Kit (Cat no. A25771, Thermo Fisher Scientific, USA) with the Ion Proton System (Cat no. 4476610, Thermo Fisher Scientific, USA) ^26^. Libraries were prepared using Ion AmpliSeq cancer HotSpot Panel v1 (Cat no. 4471262, Thermo Fisher Scientific, USA) primer pools. The Ion AmpliSeq Library Kit 2.0 (Cat no. 4475345, Thermo Fisher Scientific, USA) and Ion PI Hi-Q OT2 200 Kit (Cat no. A26434, Thermo Fisher Scientific, USA) was used for library and template preparation respectively. Sequencing was carried out using Ion PI Hi-Q Sequencing 200 Kit (Cat no. A26433, Thermo Fisher Scientific, USA) reagents and libraries were tagged with Ion Express Barcode Adapters 1-16, Cat. No. 4471250 (Thermo Fisher Scientific, USA). After sequencing, amplicon sequences were aligned to the human reference genome GRCh37 (hg19) (Accession no. GCA_000001405.1) in the target region of the cancer HotSpot panel using the Torrent Suite Software v.5.0.2 (Thermo Fisher Scientific, USA). Variant call format files (vcf files) were generated by running the Torrent Variant Caller Plugin v5.2. Variant calling and creation of vcf files can also be carried out using non-proprietary software such as SAMtools ^27^ or VarScan2 ^28^, which also provide coverage analysis. The vcf file data were analyzed using Ion Reporter v5.6 (ThermoFisher Scientific, USA), which calculated allele coverage, allele frequency, allele ratio, variant impact, clinical significance, PolyPhen 2 scores, Phred scored, SIFT scores, Grantham scores and FATHMM scores. This vcf file analysis was also carried out by Advaita Bioinformatics’ iVariantGuide. PolyPhen2, SIFT, variant impact and clinical significance can be calculated using non-proprietary software SnpEff ^29^ and SnpSift ^30^. FATHMM scores can also be predicted using fathmm ^31^ and Grantham scores according to the formula as described in Grantham, 1974 ^32^. The heat map was generated by the clustering of predicted variant impact scores by Ion Reporter v5.6. The most deleterious score was picked for every gene to generate the heat map; thereafter, hierarchical clustering was conducted. The color codes indicate the following variant impacts using score values 0-8: (0) unknown; (1) synonymous; (2) missense; (3) non-frameshift block substitution; (4) non-frameshift indel; (5) nonsense; (6) stop-loss; (7) frameshift block substitution or indel; (8) splice variant.

## Results

### Clinical presentation and radiology

**Figure 1.**
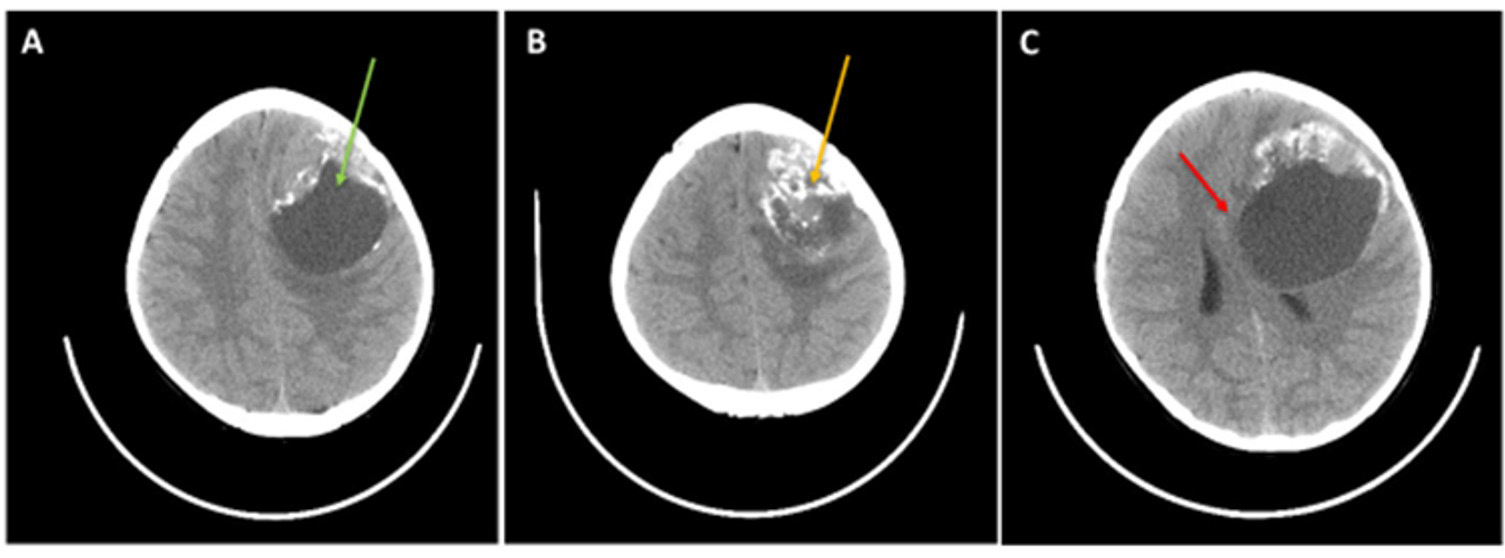
Grade III ependymoma unenhanced computed tomography (CT) of the brain. A large lesion (5.4 × 7.5cm) in the left cerebral frontoparietal location with predominantly cystic components (panel **A**, green arrow), and a large, eccentric clump of coarse calcification (panel **B**; yellow arrow). Mass effect and mid line shift (panel **C**, red arrow) can also be seen. No hydrocephalic changes or intrinsic active hemorrhagic focus were observed.

A six-year-old female patient presented with a history of right facial palsy for few months with ataxia and right-sided weakness. The patient had a chronic headache, vomiting and had repeatedly been treated for sinusitis. Unenhanced computed tomography (CT) of the brain was performed ( Figure 1, panels A, B and C). A large lesion (5.4 × 7.5cm) was noticed in the left cerebral frontoparietal region. There was an indication of a predominant cystic component and large, eccentric clump of coarse calcification. Additionally, mass effect resulting in midline shift, along with mild scalloping of the internal cortex of the parietal bone, was noted. No hydrocephalic changes or intrinsic hemorrhagic focus were seen ( Figure 1).

**Figure 2.**
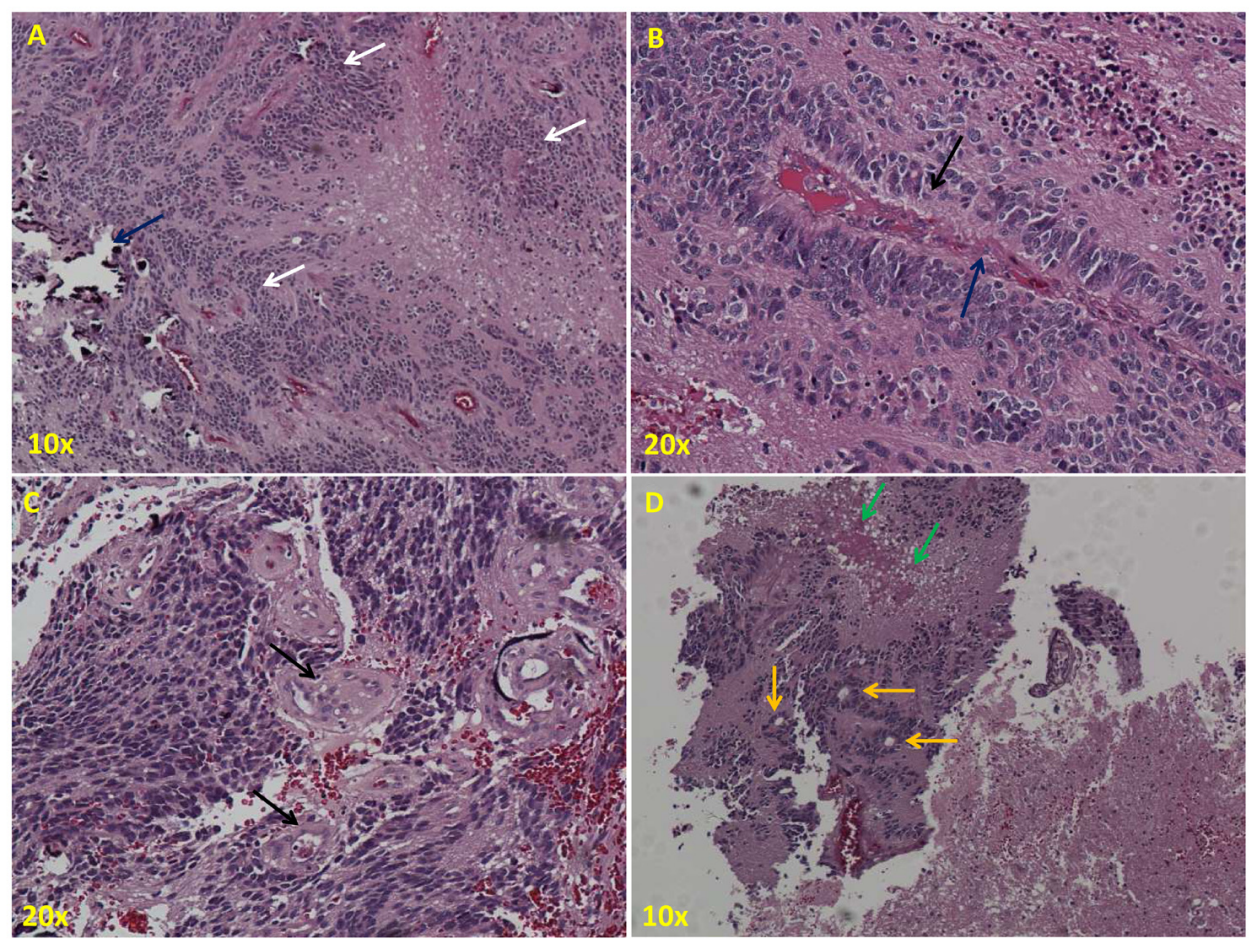
Hematoxylin and eosin (H&E) staining showing anaplastic ependymoma features. ( **A**) Focal calcification areas (blue arrow), and perivascular pseudo-rosettes (white arrow). ( **B**) Pseudo palisading necrosis, characterized by a garland-like structure of hypercellular tumor nuclei (black arrow) lining up around irregular foci of tumor necrosis (blue arrow). ( **C**) The cellular tumor exhibiting glomeruloid vascular proliferation (black arrows). ( **D**) Extensive palisading necrosis (green arrows) and true rosettes (yellow arrows).

**Figure 3.**
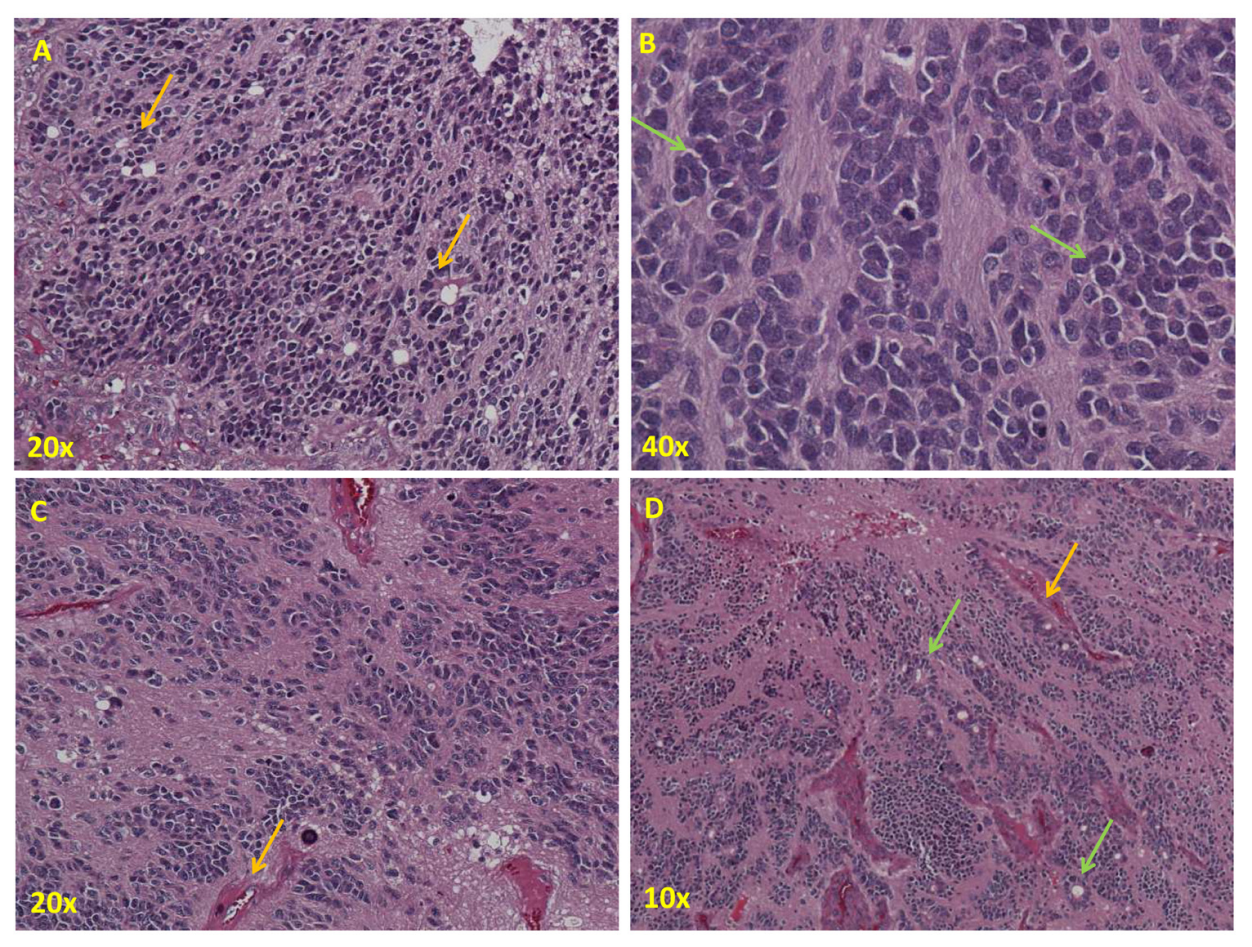
Hematoxylin and eosin (H&E) staining showing anaplastic ependymoma features. ( **A**) Pseudo palisading necrotic areas, exhibiting true rosettes with central lumen (yellow arrow). ( **B**) Focal areas with numerous tumor giant cells and the presence of a brisk mitotic activity (green arrows). ( **C**) Tumor with vascular formation (yellow arrows) and pseudo palisading necrotic areas. ( **D**) Formation of true rosettes (green arrows) surrounding the microvascular proliferation within ependymal tumors, usually signifies anaplastic transformation which is characteristic of ependymomas.

**Figure 4.**
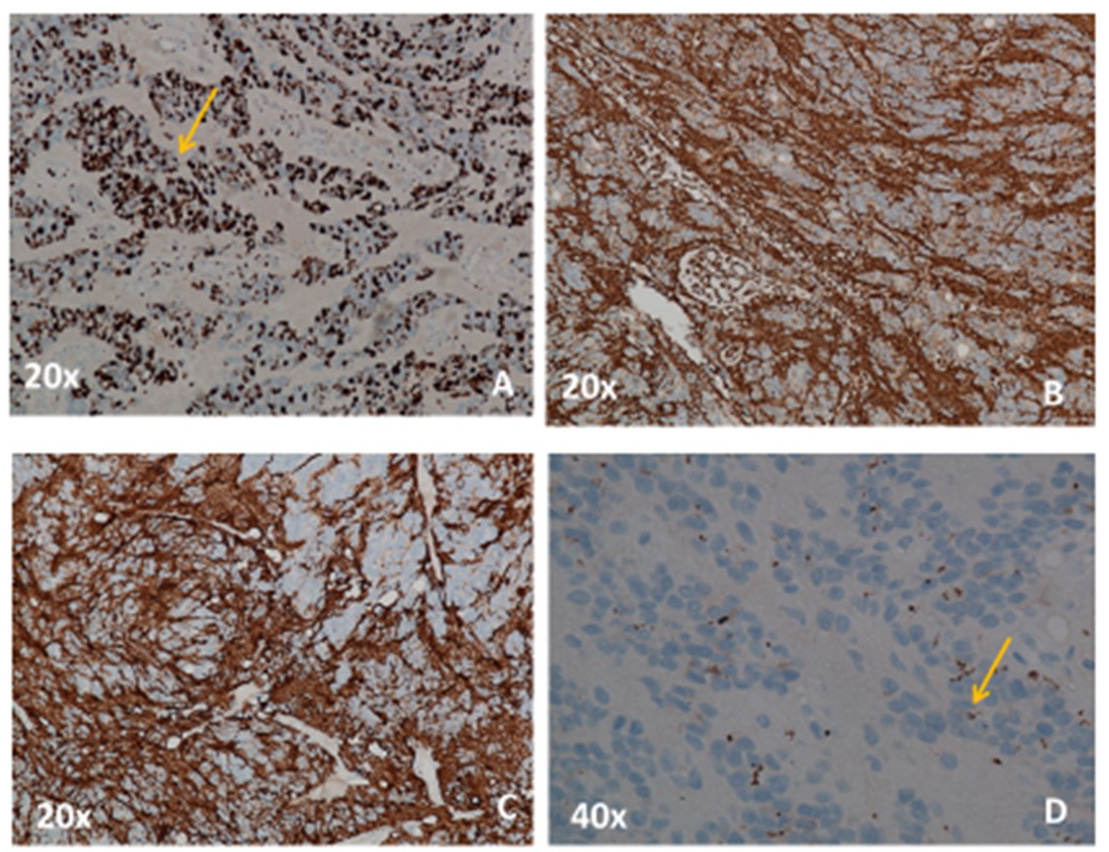
Photomicrographs of Ki-67, vimentin, GFAP, and EMA immunostaining of the ependymal tumor. ( **A**) Ki-67 immunostaining indicates a high proliferation index in the tumor (70%). ( **B**) Vimentin stain is positive. ( **C**) GFAP stain is positive. ( **D**) EMA stain is positive and shows punctate cytoplasmic (perinuclear dot-like) staining, fairly diagnostic of the ependymal nature of the tumor cells.

**Figure 5.**
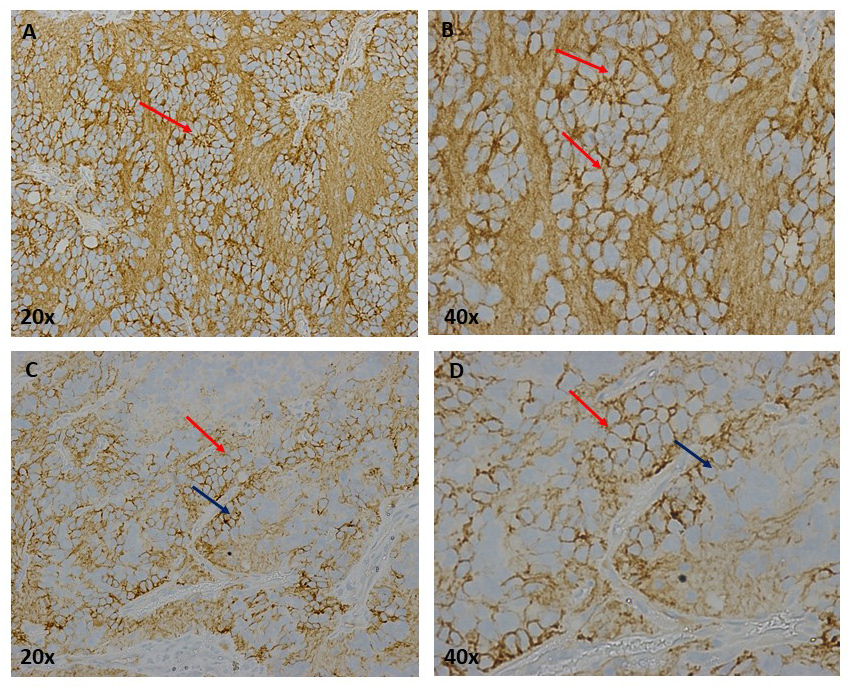
Photomicrographs of beta-Catenin and E-Cadherin immunostaining of the ependymal tumor. Immunostaining is strongly positive for beta-Catenin (panel **A** 20x, panel **B** 40x) and true rosettes (red arrows) and palisading cells (blue arrow) are clearly visible. E-Cadherin stain is also positive in this tumor. Red and blue arrows indicate tumor cells arranged in true rossettes and formation of palisading structures, respectively (panel **C** 20x, panel **D** 40x).

Histopathological examination revealed sheets of neoplastic cells with round to oval nuclei and abundant granular chromatin. A variable dense fibrillary background and endothelial proliferation was also noted. Hematoxylin and eosin (H&E) staining results are shown in Figure 2 and Figure 3. Panels A and B of Figure 2 show the tumor exhibiting delicate cytoplasmic processes, perivascular rosettes characteristic of ependymoma, focal calcification areas and pseudo palisading necrosis, characterized by a garland-like structure of hypercellular tumor nuclei lining up around irregular foci of tumor necrosis. Panel C shows glomeruloid vascular proliferation and panel D shows extensive palisading necrosis and true rosette formation. The exhibition of a true rosette with a central lumen and the formation of pseudo-palisading necrotic areas is also clear from Figure 3 (panel A). Panel B shows focal areas with numerous tumor giant cells and the presence of brisk mitotic activity, vascular formation and pseudo-palisading necrotic areas. Formation of true rosettes surrounding the microvascular proliferation within ependymal tumors usually signifies anaplastic transformation, which is characteristic of grade III ependymoma (panels C and D). Immunostaining is shown in Figure 4: (A) Ki-67 stain shows a high proliferation index, (B) vimentin positive, (C) GFAP positive, (D) EMA showing punctate cytoplasmic (perinuclear dot-like positivity) staining which is fairly diagnostic of ependymal tumor cells. Figure 5 shows beta-catenin positive (panels A and B) and E-cadherin positive (panels C and D) immunostaining, with both membranous and true rosette-like structures clearly visible in this staining. EGFR staining was negative (see *Underlying data*) ^33^.

### NGS data analysis variant identification and variant statistics

**Figure 6.**
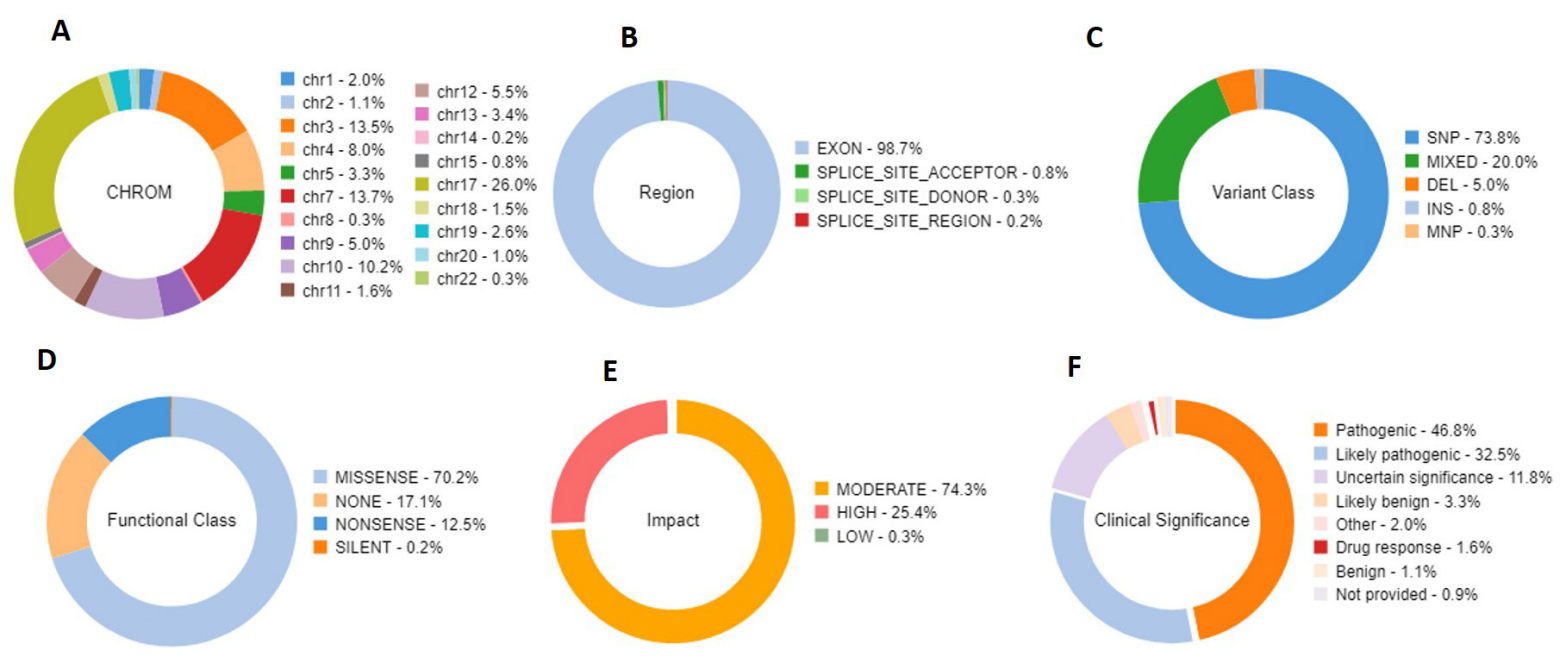
iVariant analysis of variant characteristics. Distribution of variants according to filters, showing characteristics including the relative number of variants located on each chromosome, variant class, substitution type and the functional consequences of each variant, in order to interpret and score the severity and impact of variants and therefore predict the severity of the disease. Doughnut charts in panels shows variants passed for each individual filter for ( **A**) Chromosomal distribution, ( **B**) Region in the gene, ( **C**) Variant class, ( **D**) Variant effect on the protein structure, ( **E**) Variant impact on the protein function and ( **F**) Clinical significance of the variants as annotated on the ClinVar database.

Alignment to the target regions (CHP2. 20131001.designed) of the reference genome (hg 19) was performed by the Ion Torrent Suite software v.5.0.2. For this tumor, NGS generated 6,252,341 mapped reads using the Ion PI v3 Chip, with more than 90% reads on target. Amplicon and target base read coverages for the sequencing are shown in Table 1. All 207 amplicons were sequenced with Ion AmpliSeq Cancer HotSpot Panel primer pool. As shown in Table 1, for this sample sequencing the uniformity of amplicon coverage was 95.17%, and the uniformity of base coverage on target was 94.81%. The average reads per amplicon was 34, 179, and the average target base coverage depth was 31,771. 100% of amplicons had at least 500 reads and the percentage of amplicons read end-to-end was 89.37% ( Table 1). Initial analysis by the Ion Reporter 5.6 program found that a total of 1652 variants passed all filters. Initial analysis by Advaita’s iVariantGuide software showed 100% (1633) of variants passed all filters (see *Extended data*) ^33^. The filter flags signify variants which do not meet certain criteria during variant calling. The flags refer to the quality or confidence of the variant call. The parameters of flags were read in from the input vcf file. If a variant passes all filters, it is marked as having passed. Six hundred and fifteen variants were identified using a filter for clinical significance that identifies drug response, likely to be pathogenic and pathogenic variants. The distribution of these variants, based on chromosomal position, region within the gene, variant class, functional class, variant impact and clinical significance, are shown in doughnut charts A – F ( Figure 6). As shown in doughnut chart A, chromosome 17 has the highest number of variants (26%) and chromosome 8 has lowest number of variants (0.8%). 98.7% of variants are exonic and, according to variant class distribution, 73.8% are SNPs, 70.2% are missense variants, 25.4% are high impact variants and 46.8% are pathogenic. We have considered true mutations to be those with a Phred score above 20 and significant mutations called by Ion Reporter software were those with a p-value below 0.05.

**Table 1.** Coverage analysis of the tumor DNA sequencing on Ion Proton.

Amplicon Read Coverage		Target Base Coverage	
Number of amplicons	207	Bases in target regions	22,027
Percent assigned amplicon reads	97.53%	Percent base reads on target	89.69%
Average reads per amplicon	34, 179	Average base coverage depth	31,771
Uniformity of amplicon coverage	95.17%	Uniformity of base coverage	94.81%
Amplicons with at least 1 read	100%	Target base coverage at 1x	100%
Amplicons with at least 20 reads	100%	Target base coverage at 20x	100%
Amplicons with at least 100 reads	100%	Target base coverage at 100x	100%
Amplicons with at least 500 reads	100%	Target base coverage at 500x	100%
Amplicons with no strand bias	97.58%	Target bases with no strand bias	96.35%
Amplicons reading end-to-end	89.37%	Percent end-to-end reads	86.80%

**Figure 7.**
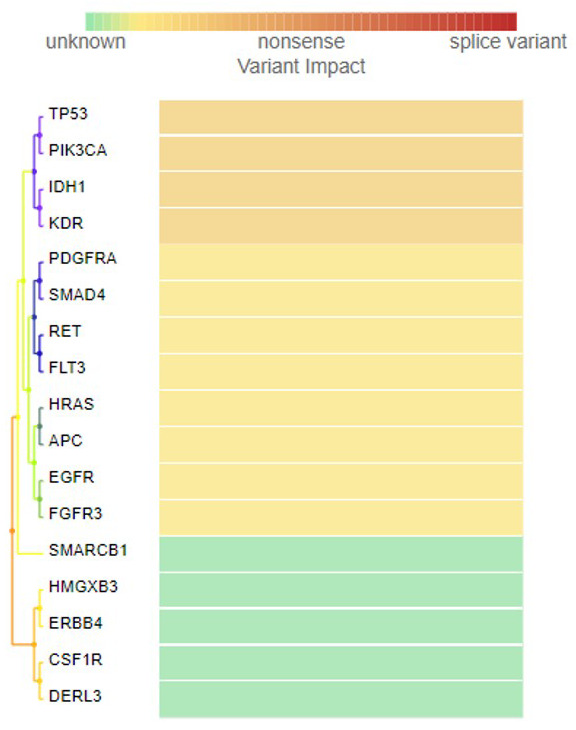
Heat Map showing variant impact of each gene detected in the ependymal tumor. Variant impact takes into account the type of mutation (such as insertion, deletion or frame shift) and considers the location of the variant (intronic or exonic). The color gradation from green to red indicates unknown, synonymous, missense, nonsense and splice variants, calculated based upon their SIFT, PolyPhen2 and Grantham scores.

A summary of the all missense mutations found in the grade III tumor is shown in Table 2. In this tumor, NGS data analysis identified 19 variants, of which four were missense mutations, eight were synonymous mutations and seven were intronic variants. Known missense mutation c.395G>A; p.(Arg132His) in exon 4 of the *IDH1* gene, c.1173A>G; p.(Ile391Met) in exon 7 of the *PIK3CA* gene, c.1416A>T; p.(Gln472His) in exon 11 of the *KDR* gene and c.215C>G; p.(Pro72Arg) in exon 4 of the *TP53* gene were found in this tumor. The frequency, allele coverage, allele ratio, p-value and Phred score for these mutations is shown in Table 3. The p-values and Phred scores were significant for all of these mutations. The frequencies of the three missense mutations, namely *PIK3CA* c.1173A>G, *KDR* c.1416A>T and *TP53* c.215C>G, were high, suggesting that these are germ line variants, whereas the *IDH1* variant frequency was low (4.81%). As shown in Table 2, eight synonymous mutations were found in this tumor, in exon 14 of *FGFR3* p.(Thr651Thr), exon 12 of *PDGFRA* p.(Pro566Pro), exon 20 of *EGFR* p.(Gln787Gln), exon 13 of *RET* p.(Leu769Leu), exon 2 of *HRAS* p.(His27His), exon 14 of *FLT3* p.(Val592Val), exon 16 of *APC* p.(Thr1493Thr) and exon 9 of *SMAD4* p.(Phe362Phe). The synonymous mutation in *FLT3* (c.1776T>C; p.(Val592Val) detected in this tumor was a novel variant, while the other variants were previously reported. Additionally, two known intronic variants were identified in *KDR* (c.798+54G>A and c.2615-36A>CA) ( Table 2). A known splice site mutation (c.1310-3T>C) at an acceptor site in *FLT3* (rs2491231) and a single nucleotide variant in the 3’-UTR of the *CSF1R* gene (rs2066934) were also identified. Additionally, in *SMARCB1* a 5’-UTR variant, and an intronic variant in *ERBB4* and *PIK3CA* respectively were found. In Figure 7, the heat map of the variant impact for each gene is presented. The color gradation from green to red indicates unknown, synonymous, missense, nonsense, and splice variants, based upon their SIFT, PolyPhen2 and Grantham scores. Only variants in four genes had a positive PolyPhen2 score (variants in *TP53, PIK3CA, IDH1* and *KDR* genes had a PolyPhen2 score of 0.083, 0.011, 0127 and 0.003, respectively). However, FATHMM scores for the prediction of the functional consequences of a variant suggest that only the *IDH1* variant is pathogenic, with a score of 0.94. As described in the COSMIC data base, FATHMM scores above 0.5 are deleterious, but only scores ≥ 0.7 are classified as pathogenic.

**Table 2.** Variants found in the grade III ependymoma tumor.

Chromosomal Position	Ref	Observed Allele	% Frequency	Gene	Coding	COSMIC/ dbSNP	AA Change	Exon
chr2:209113112	CG	TG	4.81	IDH1	c.395G>A	COSM28746	p. (Arg132His)	4
chr2:212812097	T	C	100	ERBB4	c.421+58A>G	rs839541	p.?	
chr3:178917005	A	G	47.97	PIK3CA	c.352+40A>G	rs3729674	p.?	
chr3:178927410	A	G	54.25	PIK3CA	c.1173A>G	COSM328028	p. (Ile391Met)	7
chr4:1807894	G	A	100	FGFR3	c.1953G>A	rs7688609	p. (Thr651Thr)	14
chr4:55141050	AGCCCAGA	AGCCCGGA	100.00	PDGFRA	c.1701A>G	rs1873778	p. (Pro566Pro)	12
chr4:55962545	T	TG	43.46	KDR	c.2615-36A>CA	rs34085292	p.?	
chr4:55972974	T	A	51.35	KDR	c.1416A>T	rs1870377 COSM149673	p. (Gln472His)	11
chr4:55980239	C	T	98.35	KDR	c.798+54G>A	rs7692791	p.?	
chr5:112175769	CGG	CAG	CAG=100	APC	c.4479G>A	COSM3760869	p. (Thr1493Thr)	16
chr5:149433596	TG	GA	100	CSF1R, HMGXB3	c.*1841TG>GA, c.2954_ 2955delCAinsTC	rs2066934	p.?	
chr7:55249063	G	A	71.04	EGFR, EGFR-AS1	c.2361G>A	rs1050171	p. (Gln787Gln)	20
rs1800861	G	T	100	RET	c.2307G>T	COSM4418405	p. (Leu769Leu)	13
chr11:534242	A	G	49.07	HRAS	c.81T>C	rs12628	p. (His27His)	2
chr13:28608280	A	G	53.5	FLT3	c.1776T>C	Novel	p. (Val592Val)	14
chr13:28610183	A	G	100	FLT3	c.1310-3T>C	rs2491231	p.?	
chr17:7579472	G	C	C=47.94	TP53	c.215C>G	rs1042522	p. (Pro72Arg)	4
chr18:48591923	T	C	63.63	SMAD4	c.1086T>C	rs1801250	p. (Phe362Phe)	9
chr22:24176287	G	A	52.5	DERL3, SMARCB1	c.1119-41G>A, c.*727C>T	rs5030613	p.?	4

**Table 3.** Sequencing quality of variants found in the grade III ependymoma.

Genes	Coding	Allele Coverage	Allele Ratio	p-value	FATHMM predication	Phred Score	Coverage (x)
IDH1	c.395G>A	CG=1900, TG=96	CG=0.9519, TG=0.0481	0.00001	Pathogenic	221.064	1996
ERBB4	c.421+58A>G	C=1988	C=1.0	0.00001	NA	31774.6	1988
PIK3CA	c.352+40A>G	A=1038, G=957	A=0.5203, G=0.4797	0.00001	NA	9501.82	1995
PIK3CA	c.1173A>G	A=915, G=1085	A=0.4575, G=0.5425	0.00001	Benign	11555.8	2000
FGFR3	c.1953G>A	A=1993	A=1.0	0.00001	Benign	31840.6	1993
PDGFRA	c.1701A>G	AGCCCGGATGGACATG=1941	AGCCCGGATGGACATG=1.0	0.00001	Benign	35066.6	1941
KDR	c.2615-36A>CA	T=1124, TG=864	T=0.5654, TG=0.4346	0.00001	NA	5173.27	1988
KDR	c.1416A>T	T=971, A=1025	T=0.4865, A=0.5135	0.00001	Benign	10583.1	1996
KDR	c.798+54G>A	C=33, T=1965	C=0.0165, T=0.9835	0.00001	NA	30286.5	1998
APC	c.4479G>A	CAG=1985,	CAG=1.0	0.00001	Benign	35885.9	1985
CSF1R, HMGXB3	c.*1841TG>GA, c.2954_ 2955delCAinsTC	GA=1977	GA=1.0	0.00001	Benign	31540.7	1977
EGFR, EGFR-AS1	c.2361G>A	G=579, A=1420	G=0.2896, A=0.7104	0.00001	Benign	17657.6	1999
RET	c.2307G>T	T=1996	T=1.0	0.00001	Benign	31993.7	1996
HRAS	c.81T>C	A=1018, G=981	A=0.5093, G=0.4907	0.00001	Benign	11998.4	1999
FLT3	c.1776T>C	A=929, G=1069	A=0.465, G=0.535	0.00001	NA	11295.9	1998
FLT3	c.1310-3T>C	G=1998	G=1.0	0.00001	Benign	32026.3	1998
TP53	c.215C>G	G=1038, C=956	G=0.5206, C=0.4794	0.00001	Benign	11559.2	1994
SMAD4	c.1086T>C	T=727, C=1272	T=0.3637, C=0.6363	0.00001	Benign	14836.6	1999
DERL3, SMARCB1	c.1119-41G>A, c.*727C>T	G=950, A=1050	G=0.475, A=0.525	0.00001	NA	13246.6	2000

## Discussion

Ependymomas are brain tumors that arise throughout the central nervous system, within the supratentorial areas, the posterior fossa and the spinal cord. Histologic low-grade (WHO grade I) tumors, such as subependymomas and myxopapillary ependymomas, are usually slow progressing variants of ependymomas. In contrast, grade III ependymomas display anaplastic features like hypercellularity, high mitosis, proliferation of endothelial cells and palisading necrosis ^34^. Histopathological evaluation of ependymoma tissue reveals pseudo-rosette formation, high mitotic activity, vascular proliferation and necrosis, EMA staining with perinuclear dot-like structures and with diffuse GFAP immunoreactivity ^35^. Immunological staining with GFAP and vimentin is very helpful for the differential diagnosis of ependymomas from other non-ependymal tumors, such as astrocytic and choroid plexus tumors, and also in differentiating between the various grades of ependymomas ^13, 14, 36^. It has been reported that the GFAP expression correlates with a loss of E-cadherin expression in anaplastic ependymomas, although in this case there was E-cadherin expression ^36^. Changes in E-cadherin expression promote tumor invasion and metastasis ^37^. Overexpression of EGFR is known to correlate with tumor grades in ependymomas (100%, 50%, and 0% in grade I, II and III, respectively) ^23^. The tumor in our case is grade III anaplastic ependymoma and it stained negatively for EGFR, confirming this observation. Based upon the expression profiles of numerous angiogenesis genes (HIF-1a signaling, VEGF signaling, cell migration) and signaling pathway genes (PDGF signaling, MAPK signaling, EGFR signaling), posterior fossa ependymomas are subdivided into two groups ^19^. In this case, a diagnosis of anaplastic ependymoma (WHO grade III) was made upon the observation of the above characteristics for the tumor. The pathology of the resected tissue demonstrated a hypercellular tumor with areas of perivascular pseudo rosettes, consistent with a diagnosis of ependymoma.

Despite several investigations, the correlation between histological grading of ependymoma tumors and their prognosis is unclear ^8, 34, 38^. Apart from histopathological grading, previous studies have focused on gross deletions and chromosomal abnormalities through cytogenetic studies and array-CGH profiling of ependymomas ^39, 40^. These studies helped to distinguish between intracranial and spinal cord ependymomas. Around 70% of supratentorial ependymas are known to carry a fusion gene which produces the *C11orf95/RELA* fusion transcript and the prognosis is poor for this tumor ^41^. Ion Torrent PGM sequencing of a grade II ependymoma demonstrated *MET* and *ATRX* copy number gain ^25^. Overexpression of L1 cell adhesion molecule ( *L1CAM*), 1q25 copy number gain and a homozygous deletion in *CDKN2A* was also reported in some aggressive supratentorial ependymomas ^42^. However, in the present case we did not detect any *MET* or *CDKN2A* mutations using the Ion AmpliSeq Cancer HotSpot panel. 

In the cancer genome atlas (TCGA) projects top mutated cancer genes were *IDH1*, *TP53*, *EGFR*, *PIK3CA*, and *PDGFRA* (cBioportal data base, https://www.cbioportal.org). The specific variants we found in the present ependymoma case such as in *TP53, HRAS, SMAD4, PIK3CA* are not in TCGA projects. However, genes that are mutated in this ependymoma tumor such as *IDH1*, *TP53*, and *EGFR* are in top 20 mutated cancer genes with high functional impact, other genes were not in top 20 genes in ICGC data portal ( https://dcc.icgc.org). In the integrative onco-genomics data base ( https://www.intogen.org/search?cancer) genes mutated such as, *TP53* and *PDGFRA* are in high-grade glioma data from St. Jude children's research hospital (HGG_D_STJUDE), and *IDH1, TP53, PIK3CA* and *EGFR*, are most recurrently mutated cancer driver genes in GBM_TCGA dataset. Also, under ‘ependymoma’ only search, two cohorts are found, with total 94 samples. In the ependymoma – DKFZ cohort, (EPD_PRY_DKFZ_2017), total 55 samples are found. The *IDH1* variant found in our case also detected as a mutational cancer driver in this cohort ^43^. Ependymoma data from St. Jude children's research hospital have 39 samples, and by WGS, *PIK3CA* is detected as a mutational cancer driver in the ependymoma cohort in 2 out of 39 (5.13%) samples (3:179203765: T>A; AA345, 3:179221147: A>C, AA726), were found to have mutation in this driver gene ^41^.

Patients with neurofibromatosis type 2 are predisposed to the development of ependymomas, and the gene for neurofibromatosis type 2 ( *NF2*) maps to chromosome 22 (q1216,17). Mutations in the *NF2* gene are uncommon in sporadic ependymomas and appear to be restricted to spinal tumors ^44^. For spinal cord ependymomas, four out of eight tumors were found to have an *NF2* mutation and all eight tumors had loss of heterozygosity (LOH) of chromosome 22, where the *NF2* locus is found. However, five out of eight intracranial tumors exhibited LOH of chromosome 22 but no *NF2* mutations ^24^. A high rate of truncating mutations such as nonsense and frameshift mutations in the *NF2* gene were also reported previously in spinal ependymomas ^45, 46^. Unfortunately, in the Ion AmpliSeq cancer HotSpot panel primer pool used in the present study NF2 gene was not included. The *SMARCB1* germline mutations contribute to 10% of sporadic schwannomatosis. The SNP (rs5030613) found by us in this gene c.1119-41G>A is also reported in Schwannomatosis ^47^. However, this SNP was not reported previously in ependymomas. 

We have verified all mutations in various databases (COSMIC, ExAc and dbSNP) to confirm whether variants are novel. Only one detected in our case, a synonymous variant found in *FLT3* (c.1776T>C; p.(Val592Val), is a novel variant. In 924 glioma cases tested *FLT3* mutations found in 26 cases, a mutation in Val592 codon [c.1774G>A; p.(V592I)] was reported in 2 cases of astrocytoma grade IV. In this cohort 16 ependymoma case were included but their mutation status for *FLT3* is negative ^48^. Identification of this novel variant in exon 14 of *FLT3* does not have any structural functional impact as this is a synonymous variant coding for the same amino acid. However, this variant is not reported in COSMIC database or dbSNP also. In the Leiden open variation database (LOVD) 5 more synonymous mutations were reported in exon 14 (in the juxta membrane domain amino acids 572-609); in c.1746, c.1770, c.1773, c.1803 and in codon 1815 http://databases.lovd.nl/whole_genome/variants/FLT3. The *IDH1* mutation c.395G>A; p.(Arg132His) we detected in this tumor is a substitution missense mutation which has been reported previously (COSM28746) in glioma tumors ^49^. In this codon, another missense G>T mutation (COSM28750), and a compound substitution c.394_395CG>GT (COSM28751) are also known. Somatic *IDH1* mutations in this codon have been found with greater frequency in diffuse astrocytomas, oligodendrogliomas, oligoastrocytomas and secondary glioblastomas. And in anaplastic ependymoma grade III this variant is reported with 14.3% frequency ^50^. However, several grade II and grade III ependymal tumors tested did not show this mutation in the *IDH1* gene ^51^. For astrocytic tumors, the presence of this mutation is known to be associated with younger patients ^52^. This observation supports our findings for this ependymoma tumor as the patient is six years-old. This mutation is pathogenic, having a FATHMM score of 0.94. Other variants detected in this tumor, such as those in *FGFR3*, *PDGFRA*, *KDR* (c.1416A>T), *CSF1R*, *EGFR*, *RET*, *HRAS*, *PIK3CA*, *FLT3* (c.1310-3T>C), and *SMAD4,* are benign. The *FLT3* splice variant c.1310-3T>C (rs2491231) was reported in 84% of triple negative breast cancer cases 53. This variant was not reported in ependymoma tumors previously, this is the first time we report it here. Variants detected in this tumor have also been reported in other cancers: *PDGFRA* mutations in cervical adeno-squamous carcinomas; *ERBB4* mutations in lung adenocarcinomas; *FGFR3* mutations in breast, endometrial and ovarian cancers; *CSF1R* mutations in prostate cancer; *EGFR* mutations in lung adenocarcinomas; *RET* mutations in thyroid carcinomas; *HRAS* mutations in melanomas; and *SMAD4* mutations in breast cancer. However, with the exception of the *KDR* variant c.1416A>T, this is the first time the above variants are reported in a brain tumor ^54– 62^.

We found an intronic variant in *PIK3CA* and one missense mutation in this gene. This missense mutation was also reported previously in hemangioblastoma and in colon adenocarcinoma ^63, 64^. Missense mutations in *PIK3CA* are known to promote glioblastoma tumor progression ^65^. Mutations of the *PTEN* gene are rare in ependymomas and we have also not detected any *PTEN* mutations in this tumor ^66^. The *KDR (VEGFR2)* gene plays an important role in neovascularization and tumor initiation by glioma stem-like cells ^67^. In non-small cell lung cancer patients, the Gln472His SNP is associated with increased *KDR* activity, and was correlated with increased micro vessel density ^68^. This variant was not known in ependymoma tumors previously. This mutation is reported in Colo-rectal cancer, melanoma, non-small cell lung cancer, and it’s an important target for drugs like Avastin (Bevacizumab), Aflibrcept, and drugs reported in ( http://atlasgeneticsoncology.org/Genes/GC_KDR.html).

Mutations in cancer driver genes such as *TP53*, *CDKN2A*, and *EGFR*, which are frequently affected in gliomas, have been shown to be rare in ependymomas ^44, 66, 69^. We have detected a *TP53* mutation (c.215C>G, p.Pro72Arg, rs1042522) in this tumor with a frequency of 47.94%. This mutation p.(Pro72Arg) has also been reported previously in a medulloblastoma tumor in a young patient ^70^. Previous studies have shown that out of 15 ependymoma tumors tested, only one case, a patient with a malignant ependymoma of the posterior fossa, had a mutation in exon 6 of the *TP53* gene, which was silent, and in another study only one out of 31 ependymoma tumors tested contained a mutation in the *TP53* gene ^71, 72^. However, in another study, out of 15 ependymoma tumors, none had a mutation in the *TP53* gene, suggesting that this gene does not play an important role in the pathogenesis and development of ependymomas, unlike other brain tumor types ^66, 72, 73^. Miller *et al.*, (2018) through whole-exome sequencing of an anaplastic ependymoma tumor, have shown mutations in several cancer-related genes, as well as genes related to metabolism, neuro-developmental disorder, epigenetic modifiers and intracellular signaling ^74^. These authors have shown resistance-promoting variant expression in a single ependymoma case at different stages of recurrence. However, these genes were not present in the cancer panel we used in this study. Using the human exome capture on Illumina, Bettegowda *et al.*, (2013) have reported that in one out of eight grade III intracranial ependymomas, tumors have mutations in *PTEN* and *TP53*, and one tumor with *HIST1H3C* mutations ^24^. The *HIST1H3C* p.(Lys27Met) mutation has also been reported previously in posterior fossa ependymomas ^75^. Ependymomas may in fact represent a very heterogeneous class of tumors, each with distinct molecular profiles and, even within posterior fossa ependymomas, there are at least two distinct gene expression patterns, as demonstrated by Witt *et al.*, (2011) ^19, 76^. Overall, in previous studies, a very low frequency of mutations was observed in both intracranial and spinal ependymomas and our findings also supports this observation ^19, 24, 25, 41^.

The Ion AmpliSeq Cancer HotSpot Panel consists of 207 primers in 1 tube, targeting 50 oncogenes and tumor suppressor genes that are frequently mutated in several types of cancers. The detected mutations were found to have high accuracy; 100% amplicons had at least 500 reads and 500x target base coverage was also 100%. This high level of accuracy and the high depth of coverage achieved with the Ion Proton system allowed us to reliably detect low frequency mutations with high confidence. Allele coverage in most of the variants is around 2000x, the p-value was 0.00001 and the Phred score was very high for all the variants, indicating high confidence in the variants found in this tumor. Apart from its use in whole-exome sequencing, cancer panel analysis has also become common practice for Ion Proton ^26^. The Ion Proton instrument has the advantage of pooling samples using barcodes and the Ion PI chip. For pooled samples, sequencing enables a high throughput up to 15 Gb of data, with more than 60–80 million reads passing read filtering. The purpose of read filtering is to discard the reads that contain low quality sequences, to remove polyclonal reads, remove reads with an off-scale signal, remove reads lacking a sequencing key, remove adapter dimers, and remove short reads etc. If the computed mean read length from all the reads and the minimum total mapped reads in the sample is less than the specified threshold, that sample does not pass the quality control.

Recent molecular diagnostics research had helped in subdividing glioblastomas, oligodendrogliomas and oligoastrocytomas into genetically diverse groups of tumors, and these mutational markers may help in predicting the prognosis and response to therapy ^77^. However, such a strategy for the molecular subdivision of ependymomas has been not successful so far using mutational profiling. Epigenetic markers and fusion protein analysis have also helped in identifying new groups of supratentorial ependymoma tumors and in spite of the histopathological signs of malignancy, a small set of ependymomas had a very good prognosis, suggesting that this subgroup of tumors should not be diagnosed as classic ependymomas ^78^. However, another study showed that methylation profiling did not identify a consistent molecular class within the supratentorial tumors, but successfully sub-classified posterior fossa ependymoma into two subgroups ^79^.

In conclusion, we have identified four known missense mutations, eight synonymous and seven intronic, in this grade III ependymoma. Out of these, only one mutation in *FLT3* (c.1776T>C, synonymous) is novel, and only one mutation in *IDH1* (c.395G>A, missense) is deleterious, with all other mutations benign. Many of the variants we reported here were not detected in the ependymal tumors analyzed by NGS previously. *HRAS* c.81T>C, *PIK3CA* c.1173A>G, *RET* c.2307G>T, *KDR* c.1416A>T, *APC* c.4479G>A, *EGFR* c.2361G>A and *FLT3* c.1310-3T>C variants have not been previously reported in brain tissue, as verified in COSMIC data base, although they have been reported in other tissues like lung and breast. Further studies are warranted, using NGS methods in all three grades of intracranial ependymomas to identify the genetic signatures that may distinguish between these tumors at the molecular genetic level. 

## Data availability

### Underlying data

Raw sequence reads for this tumor on Sequence Read Archive, Accession number SRP192752: https://identifiers.org/insdc.sra/SRP192752

Open Science Framework: Mutation profiling of anaplastic ependymoma grade III by Ion Proton next generation DNA sequencing. https://doi.org/10.17605/osf.io/y9sfg ^33^

This project contains the following underlying data:

- All variants before filteration.xlsx (spreadsheet of all annotated variants)
- Final Variant Calls using HotSpot filter.xlsx (spreadsheet of annotated variants called using HotSpot filter)
- TSVC_variants_IonXpress_013.vcf (file containing all variants (un-annotated) in vcf format)
- TSVC_variants_IonXpress_013.vcf.gz.tbi (file containing all variants (un-annotated) in vcf.gz.tbi format)
- heatmap-gr3 ion rep.csv (spreadsheet containing impact scores used to generate heat map)
- EGFR Figure neg.jpg (images for EGFR staining)
- Fig2A.jpg – Figur4D.jpg (raw image files used in Figure 2– Figure 5)
- Radiology Fig.1.jpg – Radiology Figure 1 (2).jpg (raw image files used in Figure 1)

Data are available under the terms of the Creative Commons Attribution 4.0 International license (CC-BY 4.0).

### Extended data

Open Science Framework: Mutation profiling of anaplastic ependymoma grade III by Ion Proton next generation DNA sequencing. https://doi.org/10.17605/osf.io/y9sfg ^33^

This project contains the following extended data:

- Gr-3 Advaiata Final report.pdf (Advaiata iVariant analysis report)

Data are available under the terms of the Creative Commons Attribution 4.0 International license (CC-BY 4.0).
