# Supplementary material for: Mutation profiling of anaplastic ependymoma grade III by Ion Proton next generation DNA sequencing
Source: F1000Res. 2020 Jun 22;8:613. Originally published 2019 May 2. [Version 2] doi: 10.12688/f1000research.18721.2 (PMC7317822; doi:10.12688/f1000research.18721.2)
Supplement: Peer review history

# Peer review: Reviewer response for version 2

**DOI:** 10.5256/f1000research.27281.r65242

**Published:** 2020-06-25

**Authors:** Luni Emdad

**Affiliations:** 1 Department of Human and Molecular Genetics, School of Medicine, Virginia Commonwealth University, Richmond, VA, USA

Authors have addressed my previous comments, no additional comments are noted.

Is the work clearly and accurately presented and does it cite the current literature?

Partly

If applicable, is the statistical analysis and its interpretation appropriate?

Yes

Are all the source data underlying the results available to ensure full reproducibility?

No source data required

Is the study design appropriate and is the work technically sound?

Partly

Are the conclusions drawn adequately supported by the results?

Partly

Are sufficient details of methods and analysis provided to allow replication by others?

Yes

Reviewer Expertise:

Cancer Biology and Molecular Oncology, Genetics, Targeted experimental therapeutics development

I confirm that I have read this submission and believe that I have an appropriate level of expertise to confirm that it is of an acceptable scientific standard.

# Peer review: Reviewer response for version 1

**DOI:** 10.5256/f1000research.20502.r58035

**Published:** 2020-02-26

**Authors:** Firoz Ahmad

**Affiliations:** 1 Research and Development Division, SRL Ltd, Mumbai, Maharashtra, India

The authors presented report on their NGS study on anaplastic ependymoma grade III by Ion Proton next generation DNA sequencing. 

 The article looks interesting, and is well written. Since this report is from a single case, a necessary correction has to be made in the material section. Also, the authors need to mention the tumor content of the FFPE block which was taken for the NGS analysis.

 I feel, the author need to cut down on the text significantly while explaining NGS data analysis and variant identification statistics. There is too much of information which is not required. The authors have already shared the coverage analysis report which is sufficient. 

 Except for IDH1 mutation, other variants are non significant and hence do not add much value clinically. Identification of *FLT3* p.(Val592Val) does not carry any meaning unless and until the authors show the functional impact of this variant.

 Overall, good article but the text needs to be significantly cut down considering this to be a single case report.

Is the work clearly and accurately presented and does it cite the current literature?

Partly

If applicable, is the statistical analysis and its interpretation appropriate?

Yes

Are all the source data underlying the results available to ensure full reproducibility?

Yes

Is the study design appropriate and is the work technically sound?

Yes

Are the conclusions drawn adequately supported by the results?

Partly

Are sufficient details of methods and analysis provided to allow replication by others?

Yes

Reviewer Expertise:

Molecular Diagnostics, Cancer Genetics, Next Generation Sequencing.

I confirm that I have read this submission and believe that I have an appropriate level of expertise to confirm that it is of an acceptable scientific standard.

# Response

**Published:** 2020-06-09

**Authors:** Mohiuddin Taher

**Affiliations:** Umm-Al-Qura University, USA

Response to 2 ^nd^ Reviewer’s Comments:

We are thankful to this reviewer (Dr. Firoz Ahmed, Research Scientist, and Senior Manager-R&D, SRL Diagnostics, SRL Limited, Goregaon (West), Mumbai, India) for the helpful suggestions and comments.  We have made the necessary changes in the manuscript following these suggestions.

Our answers to the comments are listed below.

Comment:

The article looks interesting and is well written. Since this report is from a single case, a necessary correction has to be made in the material section. Also, the authors need to mention the tumor content of the FFPE block which was taken for the NGS analysis.

Answer:

We agree with the reviewer’s suggestion about the sample size.  Regarding the single case, a correction is made in the manuscript in the Methods section.

As suggested by the reviewer, we have indicated tumor tissue content of the FFPE block used in this investigation (Methods section).

Comment:

I feel the author needs to cut down on the text significantly while explaining NGS data analysis and variant identification statistics. There is too much of information which is not required. The authors have already shared the coverage analysis report which is sufficient. 

Answer:

We agree with the reviewer’s suggestion, however, according to the journal's policy, we have described in detail manner several techniques used in this study and the results also in detail.  The NGS data analysis such as variant identification and coverage analysis will be helpful to reproduce this work and help others to understand the data who are not familiar with NGS analysis.

Comment:

Except for *IDH1* mutation, other variants are non-significant and hence do not add much value clinically. Identification of *FLT3* p. (Val592Val) does not carry any meaning unless and until the authors show the functional impact of this variant.

Answer:

Referee-1 also made a similar comment, and we agree with this reviewer’s suggestion.  We have made the necessary changes in the revised manuscript. We have identified in this study four missense variants; c.395G>A in *IDH1*, in c.1173A>G in *PIK3CA* in c.1416A>T in *KDR*, in c.215C>G in *TP53*. Other variants are synonymous and intronic ones.  We agree with the reviewer’s comments that “Identification of *FLT3* mutation p. (Val592Val) in exon 14 does not have any structural-functional impact as this is a synonymous variant coding for the same amino acid.”  However, this variant is not reported in the COSMIC database or dbSNP also.  It is a novel variant of *FLT3*, so it is important to report this variant.  In the LOVD database 5 more synonymous mutations were reported in exon 14 (amino acids 572-609, in the juxtamembrane domain); in c.1746, c.1770, c.1773, c.1803 and in codon 1805; http://databases.lovd.nl/whole_genome/variants/FLT3.  Internal tandem duplications of *FLT3* ( *FLT3*-ITD) are the most common mutations (in the juxtamembrane domain) associated with acute myelogenous leukemia (AML) and are a prognostic indicator associated with adverse disease outcome.  Activating internal tandem duplication (ITD) mutations in *FLT3* ( *FLT3*-ITD) are detected in approximately 20% of acute myeloid leukemia (AML).

# Peer review: Reviewer response for version 1

**DOI:** 10.5256/f1000research.20502.r53778

**Published:** 2019-10-17

**Authors:** Luni Emdad

**Affiliations:** 1 Department of Human and Molecular Genetics, School of Medicine, Virginia Commonwealth University, Richmond, VA, USA

In this study authors analyzed mutation patterns by next generation sequencing (NGS) in order to determine genomic changes in an anaplastic ependymoma. Authors identified one novel synonymous mutation and one deleterious missense mutation in this grade III ependymoma.

**Comments:**

1. In the clinical specimen section authors state “Specimens from all patients willing to give written informed consent and diagnosed with gliomas were eligible to be included in this study.” However, the specimen used in this study for NGS analysis was obtained from a single patient’s tumor tissue. What are the other samples and in what study they were used?
2. Figure 2A: authors indicate yellow arrow showing palisading necrosis; is this the correct location?
3. Authors conclude they identified four known missense mutations, eight synonymous and seven intronic, in this grade III ependymoma. How global these signature molecules in the context of grade III ependymoma? Can the authors add additional correlation analysis by analyzing TCGA or other bioinformatics based data?
4. Authors indicate FLT3 (c.1776T>C, synonymous) is novel, which is interesting; is this mutation reported in other cancer than ependymoma? What is the clinical and functional significance of this mutation in ependymoma progression?

Is the work clearly and accurately presented and does it cite the current literature?

Partly

If applicable, is the statistical analysis and its interpretation appropriate?

Yes

Are all the source data underlying the results available to ensure full reproducibility?

No source data required

Is the study design appropriate and is the work technically sound?

Partly

Are the conclusions drawn adequately supported by the results?

Partly

Are sufficient details of methods and analysis provided to allow replication by others?

Yes

Reviewer Expertise:

Cancer Biology and Molecular Oncology, Genetics, Targeted experimental therapeutics development

I confirm that I have read this submission and believe that I have an appropriate level of expertise to confirm that it is of an acceptable scientific standard, however I have significant reservations, as outlined above.

# Response

**Published:** 2020-06-09

**Authors:** Mohiuddin Taher

**Affiliations:** Umm-Al-Qura University, USA

We are thankful to the reviewer-1 (Dr. Luni Emdad, Assistant Professor, Human and Molecular Genetics Department, Virginia Commonwealth University, Richmond, USA) for the helpful suggestions and comments.  We have made the necessary changes in the revised manuscript following these suggestions.  Our answers to the comments are listed below.

**Comment:**

1. In the clinical specimen, section authors state “Specimens from all patients willing to give written informed consent and diagnosed with gliomas were eligible to be included in this study.” However, the specimen used in this study for NGS analysis was obtained from a single patient’s tumor tissue. What are the other samples and in what study they were used?

**Answer:**

The present study is a part of the project on mutation profiling of brain tumors in the Saudi population which is supported by a grant from Umm-Al-Qura University (Code. No. 43509008).  For this project, various gliomas such as GBM, astrocytomas, oligodendrogliomas, and ependymoma tumors (all grades) were collected.  The NGS analysis is ongoing for many other tumors. 

The NGS analysis reported here is for a single case, as suggested by this referee, we have modified this statement in the revised manuscript in the ‘Methods’ section.

**Comment:**

2. Figure 2A: Authors indicate yellow arrow showing palisading necrosis; is this the correct location?

**Answer:**

As suggested by the referee the corrections are made in this figure, and a new figure-2 and a new figure legend is added in the revised manuscript.

**Comment:**

3. Authors conclude they identified four known missense mutations, eight synonymous and seven intronic, in this grade III ependymoma. How global these signature molecules in the context of grade III ependymoma? Can the authors add additional correlation analysis by analyzing TCGA or other bioinformatics-based data?

**Answer:**

As suggested by the reviewer we have searched various databases including The Cancer Genome Atlas (TCGA) www.tcga.org, and summarize our answer to the reviewer’s comments below.  A relevant section of this summary is included in the revised manuscript.  The tumor type we described in this study is a rare tumor, and specifically, in many databases, it is not listed separately except in the ‘Integrative onco-genomics’ database (https://www.intogen.org).

In the integrative onco-genomics database (https://www.intogen.org/search?cancer) high-grade glioma and low-grade glioma, and GBM projects are listed.  Genes mutated such as TP53, and PDGFRA are in high-grade glioma data from St. Jude children's research hospital (HGG_D_STJUDE), and IDH1, TP53, PIK3CA, EGFR, are most recurrently mutated cancer driver genes in GBM_TCGA dataset (https://www.intogen.org).  Also, under ‘ependymoma’ only, two cohorts are found, with a total of 94 samples, 10,607 total mutations, and one driver gene is mutated in this.  

In the ependymoma – DKFZ cohort, (EPD_PRY_DKFZ_2017), a total of 55 Samples, mutations with driver genes found are nil, total mutations found are 534, (Grobner et al., 2018). Ependymoma data from St. Jude children's research hospital cloud, 39 samples are found, with 1 driver gene mutated, mutations-10,073.  By WGS, PIK3CA is detected as a mutational cancer driver in the ependymoma cohort.   2 out of 39 (5.13%) samples (3:179203765: T>A; AA, 345, 3:179221147: A>C, AA726), were found to have a mutation in this driver gene.  (Parker et al., 2014).

We have analyzed the TCGA (https://www.intogen.org/search?cancer) release 2020-02-01, dataset.  Tumor types such as medulloblastoma (636 samples), pilocytic astrocytoma (621 samples), GBM (571 samples), and LGG (565 samples) are included in this but not ependymoma.  Glioblastoma was the first cancer studied by TCGA in a pilot study. In the TCGA projects top mutated cancer genes in IDH1, TP53, EGFR, PIK3CA, and PDGFRA.  In the TCGA database the missense IDH1 mutation p.(R132H) affected cases are 90.07%, and VEP impact (Ensembl Variant Effect Predictor) is moderate for this, and SIFT impact is deleterious low confidence (score 0.01), and the PolyPhen impact is also possibly damaging (score 0.813).  Poor prognostic markers included genetic changes in the EGFR and PI3-kinase mutations in this group.  Among most driver mutations IDH1/2 and TP53 tumor grade remain a prognostic factor in diffuse gliomas (Draaisma et al., 2015).  The variant we found in the present ependymoma case such as in TP53, HRAS, SMAD4, PIK3CA not in TCGA.  However, in ClinVar database SMAD4 synonymous variant is reported (Accession: VCV000132693.2, Variation ID: 132693).

In cBioportal database (https://www.cbioportal.org) of whole-exome sequencing (WES) under diffuse glioma project for CNS/Brain tumors, only diffuse glioma, low-grade glioma, glioblastoma, medulloblastoma, and pilocytic astrocytoma and other brain tumors are classified, but ependymoma is not listed separately to apply the filter. As stated by the reviewer we can apply the search only globally under low-grade glioma (LGG) and glioblastoma (GBM World Health Organization grade IV)) projects, and for global mutation profiling not related to the variants, we described in our manuscript.  On this portal under CNS/brain studies merged cohort of LGG and GBM; TCGA, (Ceccarelli et al., 2016) a total of 1102 samples are present in that 812 (72.4%) cases are with mutations.

In IDH1 gene somatic mutations found in 411 samples (50.6%).  More than 90% of them are missense mutations. There is promising clinical data in patients with IDH1 codon R132-mutant glioma treated with the IDH1-targeted inhibitor Ivosidenib an FDA-approved drug in another indication.  This mutated amino acid was identified as a recurrent hotspot (statistically significant) in a population-scale cohort of tumor samples of various cancer types (Chang et al., 2016).

Samples with TP53 mutations are 322 (39.7%), there are no FDA-approved or NCCN-compendium listed treatments specifically for patients with c.215C>G mutant diffuse glioma.  Mutations in PIK3CA cases are 63 (7.8%).  While Alpelisib in combination with Fulvestrant is FDA-approved for the treatment of patients with PIK3CA-mutant ER+/HER2- breast cancer, its clinical utility in patients with PIK3CA mutations in diffuse glioma is unknown.  Mutations in EGFR cases are 87, (10.7%), laboratory data suggest that glioma cells with EGFR mutations may be sensitive to Lapatinib, an EGFR/HER2 tyrosine kinase inhibitor.  While the EGFR tyrosine kinase inhibitor Afatinib is FDA-approved for the treatment of patients with EGFR G719-mutant non-small cell lung cancer, its clinical utility in diffuse glioma patients with EGFR mutations is unknown.  PDGFRA mutations cases are 12 (1.5%), while Imatinib is NCCN-compendium listed for the treatment of patients with gastrointestinal stromal tumors (GISTs) harboring oncogenic PDGFRA alterations, its clinical utility in diffuse glioma patients with PDGFRA mutation is unknown.  KDR case with mutations are 1%, there are no FDA-approved or NCCN-compendium listed treatments specifically for patients with KDR mutant diffuse glioma.  FLT3 cases are 0.6%, while the multi-kinase inhibitor Midostaurin in combination with intensive chemotherapy is FDA-approved for the treatment of patients with FLT3 mutant acute myeloid leukemia (AML), its clinical utility in patients with FLT3 mutant diffuse glioma is unknown.  RET and MET cases are 0.6%, APC cases are 0.2%, ERBB4 cases are 0.4%, no clinical data is available for these genes.

FGFR3 mutation cases are 0.2%, while there is promising clinical data in patients with urothelial cancer harboring functionally characterized hotspot FGFR3 mutations treated with pan-FGFR-targeted inhibitors such as AZD4547, BGJ398, Debio1347 and Erdafitinib, their clinical utility in patients with FGFR3 mutant diffuse glioma is unknown.  None of the cases found to have SMARCB1, CSF1R, SMAD4, and HRAS, mutations. Whereas when we searched in a glioma cohort of 1004 samples (https://www.cbioportal.org/MSKCC, Jonsson et al., 2019) on this portal cases found to have SMARCB1, CSF1R, SMAD4, and HRAS mutations are 1.3%, 1.4%, 0.6%, and 0.2% respectively.

We have searched the database “Genomics of drug sensitivity in cancer” (cancerrxgene.org) to verify the studies targeting the signaling pathways of the four missense variants found in our studies.   Compounds such as AGI-5198, GSK690693, Foretinib, and MIRA-1 shown to sensitize in GBM and LGG cell lines in vitro, targeting IDH1, PIK3CA, KDR, and TP53 pathways respectively (Iorio et al., 2016).

ICGC (https://dcc.icgc.org) data portal 2504 cases in 6 projects were reported; 3 projects from the US, 1 each from Canada, Germany, and China respectively.  GBM-TCGA US-595 case, pediatric brain cancer-DE, -554 cases, LGG-TCGA, US, 514 cases were reported. In top 20 mutated cancer genes with high functional impact somatic mutations in 393 cases of LGG affected in IDH1, 24 cases of GBM affected in IDH1, 11 cases of pediatric brain cancer in IDH1, 229 cases of LGG in TP53, 105 cases of GBM in TP53, 42 cases of pediatric brain cancer in TP53, 10 cases of pediatric brain cancer in TP53.  13 cases of LGG in EGFR, 19 cases of GBM in EGFR.  Other genes were not in Top 20 genes.

In the NCI's Genomic Data Commons (GDC) portal ( https://portal.gdc.cancer.gov/) cases TCGA -GBM and TCGA -LGG are projects with 617, and 516 cases are reported, with mutations in 396, and 513 cases reported.  IDH1 mutations were found in 394 out of 903 cases, cases were affected with mutations in TP53 are 233, 38 cases were affected in PIK3CA; EGFR, 95 cases, PDGFR 20 cases, in 2 projects.

IDH1 c.395G>A p.(R132H) mutation (rs121913500), are reported in GBM, astrocytoma, and oligodendrogliomas in Clin variant database, #VCV000156444.  The frequency of this variant is highest (64.3%) in GBM WHO grade IV, compared with astrocytoma WHO grade II (7.14%), anaplastic oligodendroglioma WHO grade III (14.3%) and anaplastic ependymoma WHO grade III (14.3%) (Yusoff et al., 2016).   This variant also reported in AML as an adverse prognostic factor (Wagner et al., 2010).  The NCI's genomic data commons (GDC) have reported 28 mutations in 102 affected cases with KDR mutations in all TCGA GBM projects.  The expression KDR gene (vascular endothelial growth factor receptor 2 or VEGFR2) is increased in endothelial cells during tumor angiogenesis, and missense mutations cause constitutive activation of VEGFR2 in hemangioma.  Patients with infantile capillary hemangioma are known to have constitutive activation of VEGFR2 signaling and carry a germline mutation (C482R) in the KDR gene (Jinnin, et al., 2008). TP53 c.215C>G, PIK3CA c.1173A>G, c.352+40A>G, EGFR c.2361G>A not reported in GDC.

SMAD4 mutation in c.1086T>C (rs1801250) reported in breast cancer (Tram et al., 2011); HRAS mutation in c.81T>C rs12628, reported in chronic myeloid leukemia (Mir et al., 2015), PIK3CA mutation in c.352+40A>G, rs3729674 in breast cancer (Arsenic et al., 2015) ; ERBB4 mutation in c.421+58A>G, rs839541 in brain tissue (Mothersill et al., 2012),

TP53 mutations in GBM mostly point mutations that lead to a gain of function (GOF) of the oncogenic variants of the p53 protein (Zhang et al., 2018).  The TP53 variant rs1042522 we reported in the present case [(c.215C>G, p. (Pro72Arg)] was also reported in anaplastic astrocytoma grade-III (Pessoa et al., 2019), but not in ependymoma cases.  In COSMIC database this variant is reported in several types of cancers.  A mutation in Exon 5 of the TP53 gene was reported in one anaplastic ependymoma out of three cases (Tominaga et al., 1995). Whereas in our case the mutation was found in exon 4.

Targeted therapy is being studied for the treatment of childhood ependymoma and other brain tumors utilizing the genomic data.  NCI supported Clinical Trials for Ependymomal brain tumors 4 clinical trials are listed ( https://www.cancer.gov/types/brain/patient/child-ependymoma-treatment-pdq). One study is enrolling the patients for drug targets ( Carboplatin and Bevacizumab for Recurrent Ependymoma) that inhibit VEGF-promoted angiogenesis.  Based on the interesting results observed in the reported small series of patients with recurrent ependymomas treated with bevacizumab, as well as on the evidence of VEGF-promoted angiogenesis in these tumors, we designed a phase II study to test the efficacy of bevacizumab in patients with recurrent ependymoma.  Cabozantinib, a multi-kinase inhibitor of FLT3, MET, VEGFR2, and KIT, respectively, and clinical trials are undergoing for Non-Small Cell Lung Cancer, thyroid cancer, AML, and GBM treatment.

**References:**

Arsenic R, Treue D, Lehmann A, et al., Comparison of targeted next-generation sequencing and Sanger sequencing for the detection of PIK3CA mutations in breast cancer. BMC Clin Pathol. 2015; 15:20. doi: 10.1186/s12907-015-0020-6. eCollection 2015.

Chang MT, Asthana S, Gao SP, et al., Identifying Recurrent Mutations in Cancer Reveals Widespread Lineage Diversity and Mutational Specificity.  Nat Biotechnol.  2016; 34: 155-163. doi: 10.1038/nbt.3391.

Ceccarelli M, Barthel FP, Malta TM, et al., TCGA Research Network; Noushmehr H, Iavarone A, Verhaak RGW.  Molecular Profiling Reveals Biologically Discrete Subsets and Pathways of Progression in Diffuse Glioma (Ceccarelli et al., 2016).

Draaisma K, Wijnenga MMJ, Weenink B, et al., PI3 kinase mutations and mutational load as poor prognostic markers in diffuse glioma patients.  Acta Neuropathologica Communications. 2015; 3:88 DOI 10.1186/s40478-015-0265-4.

Grobner SN, Worst BC, Weischenfeldt J, et al., ICGC PedBrain-Seq Project; ICGC MMML-Seq Project. The Landscape of Genomic Alterations Across Childhood Cancers.  Nature. 2018; 555 :321-327. doi: 10.1038/nature25480.

Iorio F, Knijnenburg TA, Vis DJ, et al., A landscape of pharmacogenomic interactions in cancer. Cell. 2016; 166: 740 – 754. doi: 10.1016/j.cell.2016.06.017.

Jinnin, M, et al., Suppressed NFAT-dependent VEGFR1 expression and constitutive VEGFR2 signaling in infantile hemangioma. Nature Med. 14: 1236-1246, 2008.

Jonsson P, Lin AL, Young RJ, et al., Genomic Correlates of Disease Progression and Treatment Response in Prospectively Characterized Gliomas.  Clin Cancer Res.  2019; 25: 5537-5547. doi: 10.1158/1078-0432.CCR-19-0032.

Mir R, Ah I, Javid J, et al., Polymorphism T81C in H-RAS Oncogene Is Associated with Disease Progression in Imatinib (TKI) Treated Chronic Myeloid Leukemia Patients.  World J Oncol. 2015; 6: 321-328. DOI: 10.14740/wjon912e.

Mothersill O, Kelly S, Rose EJ, Donohoe G. The effects of psychosis risk variants on brain connectivity: a review. Front Psychiatry. 2012; 3:18. doi: 10.3389/fpsyt.2012.00018.

Parker M, Mohankumar KM, Punchihewa C, et al., C11orf95–RELA fusions drive oncogenic NF-κB signalling in ependymoma.  Nature. 2014; 506: 451–455.

Pessoa IA, Amorim CK, Ferreira WAS, et al., Detection and Correlation of Single and Concomitant TP53, PTEN, and CDKN2A Alterations in Gliomas. Int J Mol Sci. 2019; 20 (11). pii: E2658. doi: 10.3390/ijms20112658.

Tominaga T, Kayama T, Kumabe T, et al., Anaplastic ependymomas: clinical features and tumour suppressor gene p53 analysis. Acta Neurochir (Wien). 1995; 135(3-4):163-70. https://doi.org/10.1007/BF02187763.

Tram E, Ibrahim-Zada I, Briollais L, et al., Identification of germline alterations of the mad homology 2 domain of SMAD3 and SMAD4 from the Ontario site of the breast cancer family registry (CFR).  Breast Cancer Res. 2011 ;13(4): R77. doi: 10.1186/bcr2926.

Wagner K, Damm F, Göhring G, et al., Impact of IDH1 R132 Mutations and an IDH1 Single Nucleotide Polymorphism in Cytogenetically Normal Acute Myeloid Leukemia: SNP rs11554137 Is an Adverse Prognostic Factor. J Clin Oncol.  2010; 28: 2356-2364. doi: 10.1200/JCO.2009.27.6899.

Yusoff AM, Zulfakhar FN, Sul’ain MD, et al., Association of The IDH1 C.395G>A (R132H) Mutation with Histological Type in Malay Brain Tumors.  Asian Pac J Cancer Prev.  2016 ;17: 5195-5201. doi:10.22034/APJCP.2016.17.12.5195.

Zhang Y, Dube C, Gibert M Jr, et al., The p53 Pathway in Glioblastoma. Cancers (Basel). 2018 Sep 1;10(9). pii: E297. doi: 10.3390/cancers10090297.

**Comment:**

4. Authors indicate FLT3 (c.1776T>C, synonymous) is novel, which is interesting; is this mutation reported in other cancer than ependymoma? What is the clinical and functional significance of this mutation in ependymoma progression?

**Answer:**

A relevant section is added to the revised manuscript regarding this comment.  This mutation is not reported in the merged cohort of LGG and GBM-TCGA project (Ceccarelli et al., 2016). This driver gene FLT3 down-regulation is related to favorable clinical outcome in glioma patients (Liang et al., 2017).  Interestingly, several pathogenic mutations were reported at Val592 coding positions c.1774 and c.1775 in the COSMIC database but all in acute myeloid leukemia (AML).  However, the FLT3 synonymous mutation found in our ependymoma case (c.1776T>C) is within the juxta-membrane domain in exon 14, which codes the same amino acid valine p. (Val592Val), hence we don’t anticipate having any functional and clinical impact of this mutation. This variant is not reported in the COSMIC database or dbSNP also. The most common form of FLT3 mutation is in the internal tandem duplication (ITD) region of the juxta-membrane domain (AA 572-609), which occurs in 15–35% of patients with AML (Staudt et al., 2018). The presence of either a FLT3 ITD or TKD mutation may be associated with a response to the tyrosine kinase inhibitor, Midostaurin (Stone et al., 2017).  FLT3 activating mutations were predominant in AML, and types of leukemias and lymphomas also (Metzeler et al., 2016; The AACR Project GENIE Consortium). In the database of Genomics of Drug Sensitivity in Cancer, https://www.cancerrxgene.org/compound, mutations in FLT3 are associated with altered sensitivity to several drugs tested in vitro, also several clinical trials are ongoing using these compounds (Daver et al., 2019).  

**References:**

Staudt D, Murray H, McLachlan T, et al., Targeting Oncogenic Signaling in Mutant FLT3 Acute Myeloid Leukemia: The Path to Least Resistance.  Int. J. Mol. Sci. 2018, 19, 3198; doi:10.3390/ijms19103198

Liang, A., Zhou, B. and Sun, W. Integrated genomic characterization of cancer genes in glioma. Cancer Cell Int 2017; 17: 90. https://doi.org/10.1186/s12935-017-0458-y.

Daver N, Schlenk RF, Russell NH and Levis MJ.  Molecular targets for therapy Targeting FLT3 mutations in AML: review of current knowledge and evidence.  Leukemia. 2019; 33: 299–312. doi: 10.1038/s41375-018-0357-9.     

Metzeler KH, Herold T, Rothenberg-Thurley M, et al., AMLCG Study Group. Spectrum and prognostic relevance of driver gene mutations in acute myeloid leukemia.  Blood. 2016; 128: 686-698. doi: 10.1182/blood-2016-01-693879.

Stone RM, Mandrekar SJ, Sanford BL, et al., Midostaurin Plus Chemotherapy for Acute Myeloid Leukemia with a FLT3 Mutation.  N Engl J Med.  2017; 377: 454-464. doi: 10.1056/NEJMoa1614359.

The AACR Project GENIE Consortium. AACR Project GENIE: powering precision medicine through an international consortium. *Cancer Discovery*. 2017; 7: 818-831. DOI: 10.1158/2159-8290.CD-17-0151.
